# Supplementary material for: Content-rich biological network constructed by mining PubMed abstracts
Source: BMC Bioinformatics. 2004 Oct 8;5:147. doi: 10.1186/1471-2105-5-147 (PMC528731; doi:10.1186/1471-2105-5-147)
Supplement: Additional File 5 — The original Chilibot query results of the term "long-term potentiation (LTP)" and 22 other terms, limiting the latest references analyzed to the years 1990, 1995, 2000, and 2004. [file 1471-2105-5-147-S5.bz2 › chilibotAdditionalFile5/ltp2000/html/ARC_ACTIN.html]

 


 **ARC** and **ACTIN** 
  
Found 22 abstracts in PubMed,  **22 abstracts were retrieved and analyzed**.  


---

 Search Google  |
 PDF files only 
|  EDU domain only 

---

**Interactive relationship** (e.g. stimulation, inhibition, etc)

- An important structural feature of these cells is an  **actin**  containing  **arc**  like band on the periphery of lamella.  Ref: 7197563 Biull Eksp Biol Med, 1981
- The numerous microvilli that cover the apical plasma membrane  **arc**  supported by bundles of  **actin**  microfilaments and associated  **actin**  binding proteins.  Ref: 9019661 Nephrologie, 1996
- The one dimensional PAGE protein profile of this  **ARC**  was similar to those of Dictyostelium and Acanthamoeba  **actin**  gels.  Ref: 1340274 Arch Med Res, 1992
- Members of the Arp2 3 complex, p34  **Arc**  and p21  **Arc** , heavily concentrated in the early accumulations of F  **actin** , as did one member of the Ena VASP family Mena but not another VASP.  Ref: 10797548 J Neurosci Res, 2000
- Basic fibroblast growth factor bFGF which restricts myofibrillar growth and upregulates alpha sm  **actin**  in  **ARC**  cultured with normal FCS had no effect on alpha sm  **actin**  in  **ARC**  cultured in stripped FCS, but potentiated the effect of T3.  Ref: 8878423 J Clin Invest, 1996
- The human complex consists of seven subunits which include the  **actin**  related proteins Arp2 and Arp3, and five others referred to as p41  **Arc** , p34  **Arc** , p21  **Arc** , p20  **Arc** , and p16  **Arc**  p omplex.  Ref: 9230079 J Cell Biol, 1997
- In addition to  **actin** , the  **ARC**  was enriched with proteins that showed cross reactivity to antibodies to alpha actinin and the 50K  **actin**  binding protein elongation factor 1 alpha from Dictyostelium.  Ref: 1340274 Arch Med Res, 1992
- Arp3, p34  **Arc** , and p21  **Arc**  were localized to the lamellipodia of stationary and locomoting fibroblasts, as well to Listeria monocytogenes assembled  **actin**  tails.  Ref: 9230079 J Cell Biol, 1997
- The polarity of  **actin**  filaments was almost uniform, with barbed ends forward throughout most of the lamellipodia but mixed in  **arc**  shaped filament bundles at the lamellipodial cell body boundary.  Ref: 9334344 J Cell Biol, 1997
- During lateral expansion, the circumferential bundles of  **actin**  filaments, characteristic of IAR 2 cells, disassembled at the site of stable contact forming a concave  **arc**  like  **actin**  bundle between adjacent cells at the expanding edge.  Ref: 9023350 Proc Natl Acad Sci U S A, 1997
- Consistently, treatment of infected cells with latrunculin B, which abrogated  **actin**  filament formation, did not affect association of ActA with p21  **Arc**  at the bacterial surface.  Ref: 10954425 J Cell Sci, 2000
- In parallel, the number of alpha sm  **actin**  immunoreactive cells increased from 1% in controls to 29 and 62% in  **ARC**  treated with 5 and 100 nM T3.  Ref: 8878423 J Clin Invest, 1996

**Parallel relationship** (e.g. studied together, co-existance, homology, etc.)

- Contractile  **arc**   **actin**  filaments were revealed to be crosslinked by thin strands by the rapid freezing deep etching replication technique.  Ref: 3413069 Proc Natl Acad Sci U S A, 1988
- Retention of  **Arc**  proteins at preferred sites of  **actin**  polymerization did not require polymerization itself.  Ref: 10797548 J Neurosci Res, 2000
- However, both EGF and insulin stimulated the accumulation of phospholipase Cgamma1 at the  **actin**   **arc** , which was coincident with the EGF receptor in the case of insulin stimulated cells.  Ref: 10947866 Immunol Cell Biol, 2000
- From electron microscopic observation of thin sections of the furrow, it was suggested that the  **actin**  bundles of the contractile  **arc**  were organized from preexisting cortical filaments that were connected to the plasma membrane by granular materials at their barbed ends.  Ref: 3413069 Proc Natl Acad Sci U S A, 1988
- Therefore, it is suggested that the insulin induced coclustering of the EGF receptor with phospholipase Cgamma1 at the  **actin**   **arc**  may allow for greater efficiency of signal transduction, resulting in the synergy observed for these two hormones in stimulation of DNA synthesis.  Ref: 10947866 Immunol Cell Biol, 2000
- Besides stress fibers,  **arc**  like  **actin**  bundles have also been detected in spreading cells.  Ref: 6541223 J Cell Biol, 1984
- It was found that insulin effectively induced the accumulation of EGF receptors on the  **actin**   **arc**  of cells without activation of the EGF receptor.  Ref: 10947866 Immunol Cell Biol, 2000
